# Anatomical and Functional Study of the Ostrich (*Struthio camelus*) Lung through Macroscopic Analysis in Combination with Optical and Electron Microscopy Techniques

**DOI:** 10.3390/ani14020316

**Published:** 2024-01-19

**Authors:** Andrew Makanya, Valentin Djonov

**Affiliations:** 1 Department of Veterinary Anatomy and Physiology, Riverside Drive, Chiromo Campus, University of Nairobi, Nairobi P.O. Box 30197-00100, Kenya; 2 Institute of Anatomy, University of Bern, Baltzerstrasse 2, 3000 Berne, Switzerland; valentin.djonov@ana.unibe.ch

**Keywords:** epithelial plates, air capillaries, type II cells, secondary bronchi, ostrich lung

## Abstract

**Simple Summary:**

Ostrich is increasingly becoming an important livestock due to its high-quality products, especially its healthy meat. We studied the morphology of the ostrich lung using various imaging techniques in order to understand how it functions. The major conducting intrapulmonary airways, the secondary bronchi, were superficially placed in close proximity to the intercostal muscles and had thin collapsible external walls, implying their plausible role in gas propulsion. Many attributes of the bronchi, including categories, numbers, and topographical arrangement, were comparable to those of the Chicken. The paleopulmonic region of the lung was better developed than the neopulmonic one, which appeared rudimentary. Adjacent parabronchi were not delineated by connective tissue septa as is the case in the archetypical avian lung, and the exchange tissue was only interrupted where conducting blood vessels occurred. The parabronchi were lined with shallow atria, and, in many cases, infundibulate were absent. Air capillaries formed the terminal gas exchange units in most cases, but occasionally atria were the terminal units. Air capillaries were associated with secretory cells, and blood capillaries were supported by epithelial plates.

**Abstract:**

The Ostrich occupies a unique position as the largest bird on the planet. Like other ratites, it has been reputed to have a phylogenetically primitive lung. We used macroscopy, light microscopy, transmission and scanning electron microscopy as well as silicon rubber casting to elucidate the functional design of its lung and compare it with what is already documented for the avian species. The neopulmonic region was very small and poorly developed. The categories of the secondary bronchi (SB) present and their respective numbers included laterodorsal (8–10), lateroventral (4–5), medioventral (4–6) and posterior (16–24). The lateral aspects of the laterodorsals were covered with a transparent collapsible membrane internally lined with a squamous to cuboidal epithelium. The bulk of these SB were in close proximity to intercostal spaces and the intercostal muscles and were thought to be important in the propulsion of gases. The lung parenchyma was rigid, with the atria well supported by septa containing smooth muscles, connective tissue interparabronchial septa were absent, and blood capillaries were supported by epithelial bridges. There were two categories of epithelia bridges: the homogenous squamous type comprising two leaflets of type I cells and the heterogeneous type consisting of a type I pneumocyte and type II cell. Additional type two cells were found at the atrial openings as well as the walls of the infundibulae and the air capillaries. The atria were shallow and opened either directly into several air capillaries or into a few infundibulae. The presence of numerous type II cells and the absence of interparabronchial connective tissue septa may imply that the ostrich lung could be capable of some degree of compliance.

## 1. Introduction

The Ostrich (*Struthio camelus*) is the largest of all extant birds and belongs to the Ratite group that also includes the Emu (*Dromaius novaehollandiae*), Cassowary (*Casuarius casuarius*), Rhea (*Rhea americana*) and Kiwi (*Apteryx australis*). It is an important animal in many livestock industries because of its healthy red meat and skin [1]. The successful growth and reproductive performance of the ostrich are dependent on good nutrition and the ability of the bird to utilize semi-arid conditions in its natural habitat. The ratites are considered to be among the most primitive extant avian species [2], and they lack the distinct neopulmonic region that characterizes more phylogenetically advanced species. Ostriches are becoming increasingly important species as domestic animals, providing high-quality protein as well as non-consumable items. Furthermore, the ostrich’s embryonated egg is gaining traction as an important model for varied research [3,4], especially cancer pathogenesis [5]. There is little information published on the morphology of the ostrich lung. The only detailed investigation was based on one individual animal, and as such does not provide adequate details regarding the numbers and topography of the secondary bronchi [6]. Nonetheless, the structure of the ostrich lung was seen to generally conform to that of the other avian species. The air sacs in the ostrich are capacious and well developed, and the numbers are comparable to those of other avian species [7].

Some investigations have highlighted some salient gaps in the literature relating to the avian lung [8,9]. It would, for example, be interesting to find out whether the newly described category of secondary bronchi is characteristic of one of the most primitive avian species and whether the spatial disposition of the secondary bronchi has similarities among species [8,9,10]. As reported elsewhere, the mediodorsal secondary bronchi (MDSB) in the ostrich are superficially located, making them easily accessible for sampling respiratory gases and experimental investigations of processes such as airflow dynamics [3]. The disposition of the secondary bronchi in the duck lung [11] appears to conform to that of the Chicken (*Gallus gallus variant domesticus*) but the situation in the Ostrich has not been clarified.

An investigation of the ostrich lung parenchyma using 3D reconstruction of histological sections revealed that atria were shallow, infundibulae were few, and the majority of the air capillaries emanated directly from the atria [12]. Lack of connective tissue-based interparabronchial septa, structural features that epitomize the lungs of most highly derived metabolically active volant birds, was observed in the latter study. The complexity of the avian lung has been highlighted before [13,14,15], with the notion that its structural organization continues to draw controversy. In 3D reconstructions of the parabronchial unit, it was revealed that the terminal gas exchange units in the avian lung are not straight tubular structures but rather rotund structures that interconnect with their cognates [14]. The problem in the latter studies has been confounded by the limitations of the method since only a limited number of sections could be computed. Additionally, a 3D reconstruction based on larger amounts of sections [16] does not adequately discriminate the secondary bronchi since some categories have dimensions similar to those of parabronchi [9,10]. Failure by contemporary investigators to recognize the fifth category of secondary bronchi, namely the posterior secondary bronchi, or even failure to recognize the new nomenclature of the secondary bronchi [17] has further fueled the confusion, with some very recent publications using the old names, such as ventrobronchi [18].

It was reported earlier that incorporating several visualization techniques, coupled with study in both adult and developing subjects, greatly improves the understanding of the structure of the avian lung [19]. The current study aims to elucidate both the three-dimensional organization of the air conduits in the ostrich lung with an insight into the functional organization of the gas exchange unit.

## 2. Materials and Methods

### 2.1. Experimental Animals

Adult ostrich specimens and live ostrich chicks were obtained from Maasai Ostrich Farm, Kajiado, Kenya. Adult ostriches identified for slaughter were sexed at the premortem stage. Lung specimens only became available after the slaughtering process was completed. 

Ostrich chicks (12–21 days old) were obtained as individuals identified for culling due to leg deformities. The chicks were killed with an overdose of sodium pentobarbitone, and subsequent processing was performed as outlined below. The numbers, categories, and study procedures applied to the ostriches and ostrich chicks are given in Table 1 below.

### 2.2. Tissue Fixation

Entire ostrich rib cages intact with lungs (normally discarded at slaughter) were isolated, and lungs were filled with fixative through the trachea (either 5% formaldehyde or 2.5% glutaraldehyde in phosphate buffer). Ostrich chicks (21 days old) were obtained as individuals identified for culling due to leg deformities. The chicks were killed with an overdose of sodium pentobarbitone, and subsequent processing was performed as outlined below. Lungs intended for microscopy were fixed in situ via intratracheal infusion with a solution of 2.5% glutaraldehyde in 0.1 M cacodylate buffer (pH 7.4, 350 mOsm) and subsequent immersion into the same fixative.

### 2.3. Macroscopic Observations

**Figure 1:**
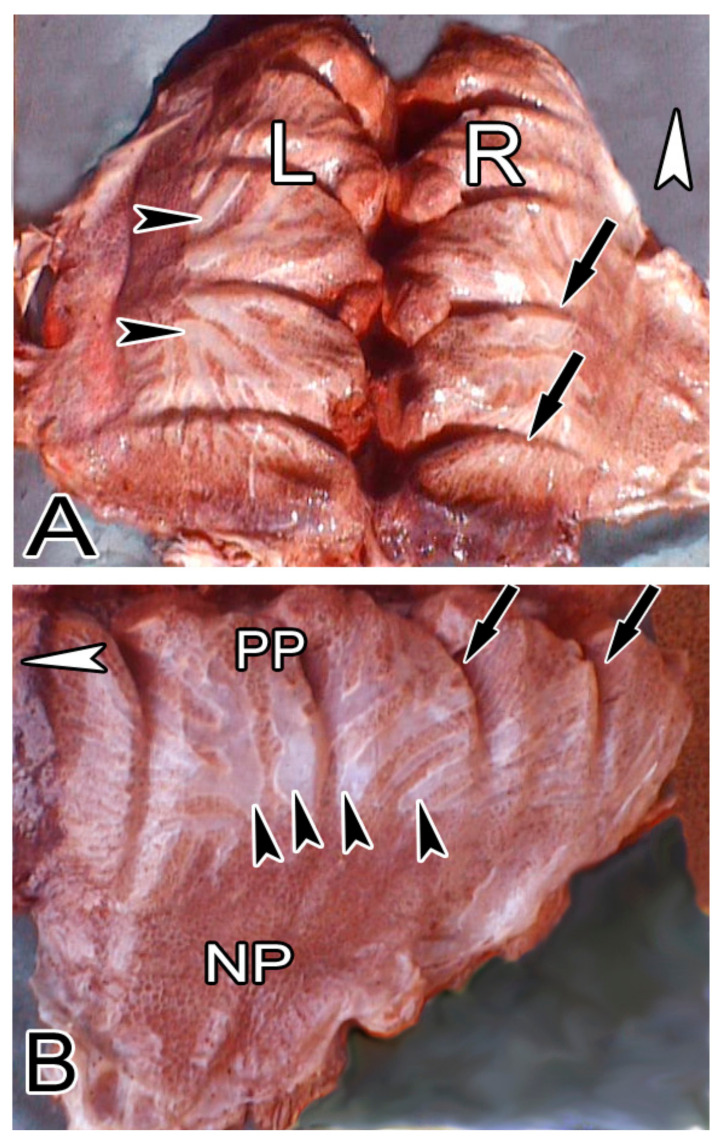
Macroscopic pictures showing the dorsal view of both lungs (**A**) and the lateral view of the left lung (**B**). The left lung (L) is a mirror image of the right one (R). Notice the prominent costal sulci (black arrows) and the transparent branches of the laterodorsal secondary bronchi (black arrowheads). The well-developed paleopulmonic region (PP) and the poorly formed neopulmonic region (NP) are shown. White arrowheads in both cases denote the cranial direction.

The lungs were carefully dissected out of the rib cage. After a general observation, a longitudinal cut was made on the ventral aspect of the lung along the long axis of the course of the mesobronchus, thus revealing the internal aspect with openings to the secondary bronchi (see Figure 1).

The identification and enumeration of the various categories of secondary bronchi emerging from the primary bronchus was conducted by counting their openings in the mesobronchus. The categories were recognized by their size and location on the wall of the mesobronchus.

### 2.4. Light and Transmission Electron Microscopy

Fixed lungs were sliced into slabs and then diced into small blocks. Tissue blocks were post-fixed in osmium tetroxide, block-stained with uranyl acetate, dehydrated through ascending concentrations of ethanol, and embedded in epoxy resin. Semithin sections were obtained at a nominal thickness of 1 μm, stained with toluidine blue, and viewed under a Leica DMBR digital light microscope. Ultrathin sections were obtained at 90 nm, counterstained with lead citrate, and viewed on a Philips CM-12 transmission electron microscope (FEI, Hillsboro, OR, USA).

### 2.5. Silicon Rubber Casting 

Silicone rubber was mixed with silicone oil at a ratio of 5:2 (volume per volume, *v*/*v*). The resulting mixture was mixed with hardener at the ratio of 1:35 (hardener to mixture, *v*/*v*). Blue dye was then added and stirred until the required color was obtained. The mixture was then injected into the tracheae of 12-day-old ostrich chicks under deep barbiturate anesthesia (Euthatal). The silicone was left to set for at least 15 min, and the lungs were dissected out and corroded in 15% potassium hydroxide. Photographs of the required parts of the resulting silicone casts were obtained with a digital camera.

### 2.6. Scanning Electron Microscopy 

The lungs were fixed via intratracheal infusion of glutaraldehyde fixative, as outlined above. Selected lung specimens were dehydrated through ascending concentrations of ethanol, critical point-dried in liquid carbon dioxide, and mounted on aluminum stubs. The specimens were sputter-coated with gold and viewed under a Philips XL 30 FEG scanning electron microscope.

## 3. Results

### 3.1. Macroscopic Observations

**Figure 2:**
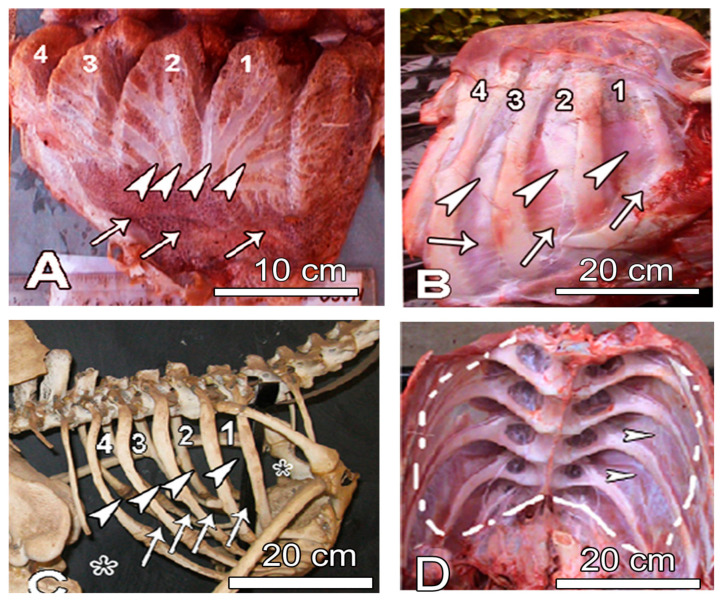
Macroscopic pictures showing the dorsal view of left lung (**A**) and the lateral view of the intact rib cage together with musculature (**B**), mounted rib cage (**C**) and the internal aspect of the intact ribcage (**D**). A: The bulk of the laterodorsal secondary bronchi (LD) are in the regions corresponding to the intercostals spaces (see arrowheads and numbers 1–4 in (**A**–**C**)). The space occupied by the lung within the ribcage is denoted by the hatched white line in (**D**). Arrows in (**A**–**C**) correspond to the intercostal parts of the neopulmonic region. White arrowheads in (**B**,**D**) point to intercostal muscles. In (**C**), the numbers 1–4 correspond to the space occupied by the lung tissue in the paleopulmonic region that is deeply entrenched between the ribs; the white arrowheads indicate the positions at which the LDSB assume a superficial position while the white arrows indicate the intercostal spaces corresponding to the lung tissue in the neopulmonic region. Asterisks denote the spaces occupied by the air sacs.

The gross appearance of the adult ostrich lung and its topographical association with the rib cage and intercostal muscles is demonstrated in Figure 1 and Figure 2. The left and right lungs appear to be mirror images of one another; lobations are absent. The lung has a somewhat rhomboid shape with a sharp ventral border and a blunt dorsal border with deep costal sulci, while the medial surface was generally smooth and flat. On the laterodorsal aspect of the lung, the laterodorsal secondary bronchi (SB) and their branches were superficially positioned just below the visceral pleura and were, as such, discernible and covered by a thin, transparent collapsible membrane (Figure 1 and Figure 2).

The positions of these SBs were such that they occupied the intercostal spaces in close proximity to the intercostal muscles. Close inspection of the fresh rib cage clearly demonstrates the intercostal furrows where the lung is entrenched adjacent to the muscles (Figure 2). This relationship is clearly captured by identifying the intercostal spaces on fresh lung specimens (Figure 2A), on fresh rib cages (Figure 2B,D) and on the skeleton (Figure 2C). 

**Figure 3:**
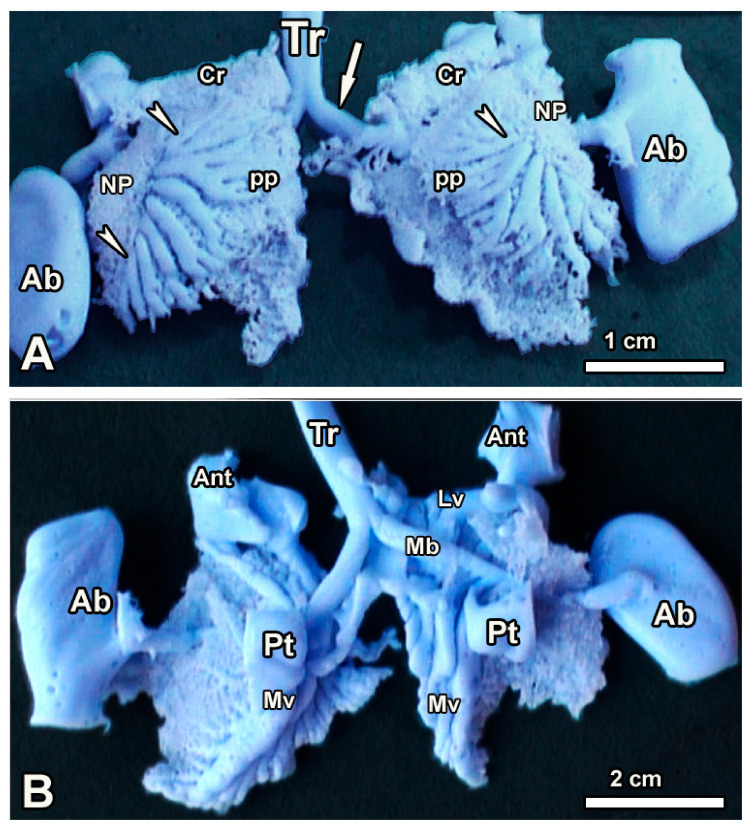
Photographs of silicon rubber casts showing the various categories of air conduits on the dorsolateral aspects (**A**) and medioventral views (**B**). The trachea (Tr) bifurcates to form the primary bronchi (white arrows) which on entering the lung tissue become the mesobronchi. White arrowheads denote the LD secondary bronchi; the poorly developed neopulmonic region (NP) and the better developed paleopulmonic regions (PP) are also shown. Some of the air sacs that have been captured are the cervical (Cr), posterior thoracic (Pt) and the abdominal (Ab). Notice the lateroventral (LV) and the medioventral (MV) secondary bronchi. This cast was made from a 12-day-old ostrich chick.

**Figure 4:**
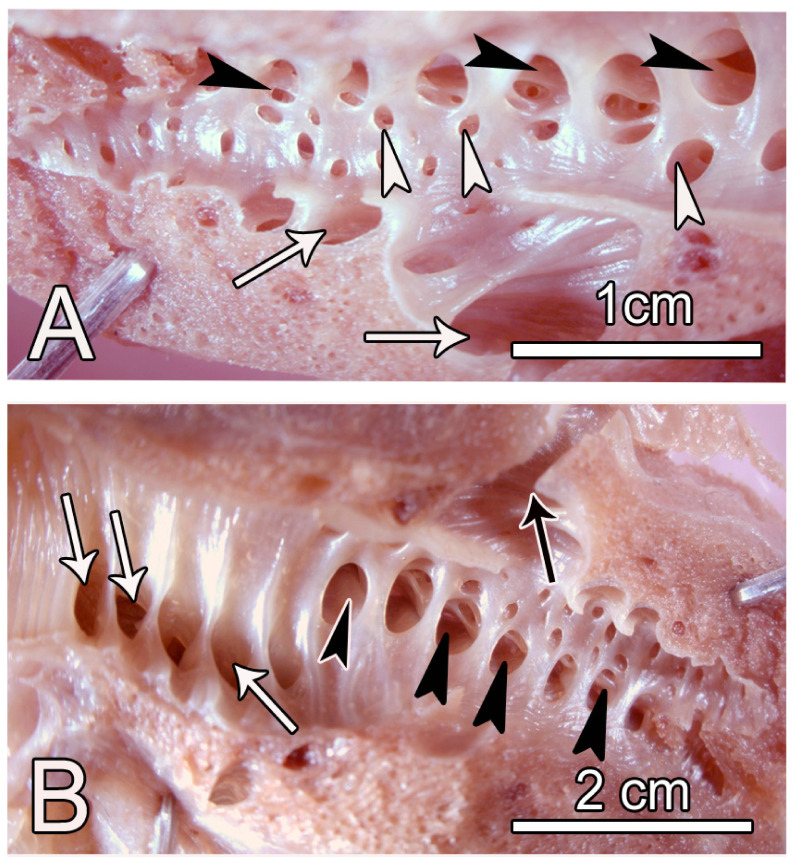
Gross specimens of a 3-week-old chick lung opened to show the positions of the various secondary bronchi. (**A**) Right lung and (**B**) left lung. The medioventral secondary bronchi (white arrows) are ventrally located at the cranial aspect of the lung. The laterodorsal secondary bronchi emerge from the mediodorsal aspect of the mesobronchus (black arrowheads), turn laterally and their bulk is found on the dorsolateral aspect. The black arrow shows one of the lateroventral secondary bronchi. The posterior secondary bronchi are the most numerous and occupy the rear two thirds of the mesobronchus (white arrowheads).

Examination and enumeration of the various categories of secondary bronchi were carried out both on silicon rubber castings (Figure 3), where the air sacs were also captured, as well as on opened fixed lungs (Figure 4), where the secondary bronchi were counted using their openings to the mesobronchus. Categories of the secondary bronchi present included laterodorsal (LDSB, 8–10), lateroventral (LVSB, 4–6), medioventral (MVSB, 4–5), and posterior (POSB, 16–24), as depicted in Table 2.

The numbers and categories of the secondary bronchi in the ostrich compare well with those of Domestic fowl and Domestic Duck (Table 3 below).

The medioventral secondary bronchi emerged from the cranial medial aspect of the mesobronchus (Figure 4) and ran medially on the ventral aspect of the lung (Figure 3). The laterodorsal secondary bronchi emerged from the mediodorsal aspect of the mesobronchus, turned laterally, and their bulk was found on the dorsolateral aspect. The posterior secondary bronchi were the most numerous and the smallest in size, occupying the rear two-thirds of the mesobronchus, and their distribution was on all sides of the mesobronchus (Figure 4).

The LVSB emerged from the lateral aspect of the mesobronchus to continue laterally, giving rise mainly to parabronchial branches that anastomosed with others in the neopulmonic region. The neopulmonic region was poorly developed and occupied a small portion of the ventrolateral aspect of the lung (Figure 3).

### 3.2. Microscopic Observations

**Figure 5:**
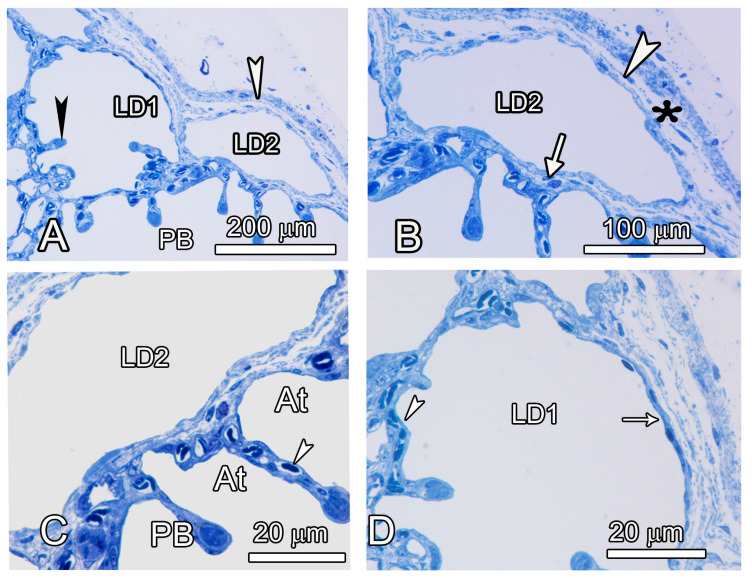
Semithin micrographs elucidating the structure of the laterodorsal secondary bronchi. (**A**,**B**) The outer wall of the terminal part of the leterodorsal secondary bronchus (LD1) lacks gas exchange tissue, but the inner aspect has atria separated by interatrial septa (black arrowheads). Other parts of the LDSB (LD2) have no exchange tissue, and both the internal and external walls are lined by non-exchange epithelium. Externally, the LDs are covered by the visceral pleura (white arrowheads in (**A**,**B**)) and in between the epithelium and the pleura is a thin layer of connective tissue (asterisks). (**C**,**D**) The inner wall of the LD2 has no exchange capillaries but it borders a large parabronchus (PB) with atria well-endowed with exchange capillaries (arrowheads). (On the external aspect, the LDSB (LD1) is lined by low cuboidal epithelium (white arrow) in (**D**).

The structure of the LDSB with collapsible membranes was followed at the light microscopic level (Figure 5); the superficial ones were lined by a cuboidal-to-squamous epithelium, had a large lumen, and were adjacent to parabronchi, with openings into the atria. The tips of such SB, however, were invaded by shallow atria on the visceral aspect with exchange capillaries. Some such atria appeared to be the ultimate gas exchange units since they did not continue to form infundibulae or air capillaries; the interatrial septa had central exchange capillaries (Figure 5). Like in other birds, the interatrial septa were reinforced with smooth muscles at the tip.

**Figure 6:**
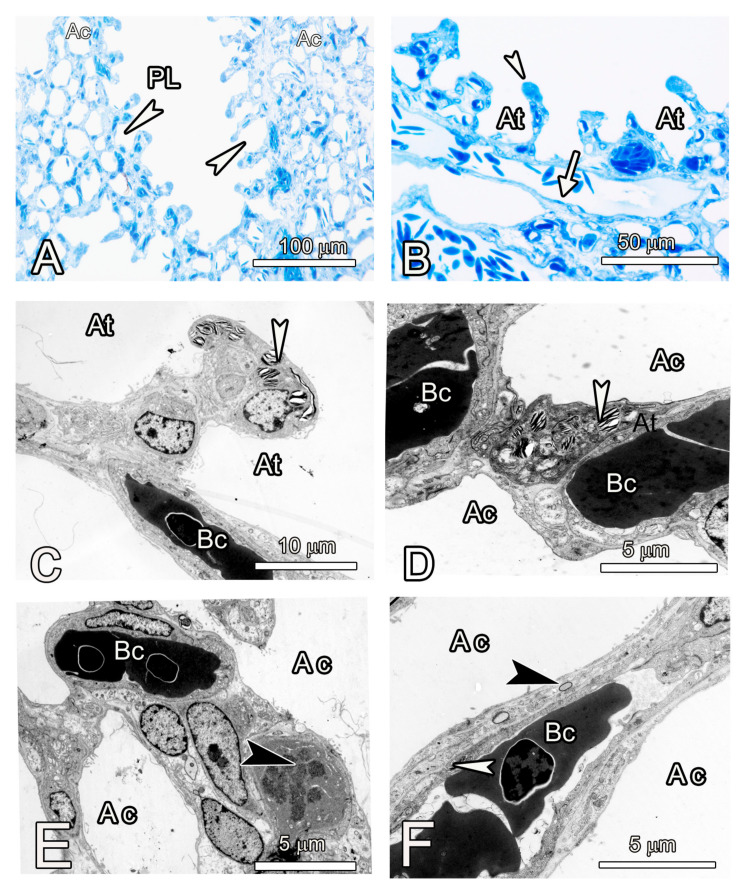
Semi-thin sections (**A**,**B**) and TEM micrographs of the gas exchange tissue of the ostrich lung (**C**–**F**). (**A**,**B**) The parabronchus (PL) is lined by shallow atria (white arrowheads) in (**A**); (At) in (**B**) that open directly into air capillaries (Ac). Notice a large blood vessel (white arrow in (**B**) below the atria, (**C**,**D**). At higher magnification, the septum separating contiguous atria (At) has a prominent type II cell at the tip while an exchange capillary (Bc) is encountered at the base of the atrium. At the air capillary level (**D**), a prominent type II cells (white arrowhead) is seen forming an epithelial bridge between two air capillaries (Ac). The bridge supports two exchange capillaries (Bc). (**E**,**F**) Mitotic figures (black arrowhead in **C**) adjacent to exchange capillaries (Bc) at the air capillary (Ac) level are thought to be type II cells undertaking cell renewal. A thin, tripartite blood-gas barrier comprising a type I cell (black arrowhead) separated from the endothelium (white arrowhead) by a thin basement membrane forms the interface between the air in the air capillary and the erythrocytes in the blood capillary (Bc).

A survey of the parenchyma at the light microscopic level revealed shallow atria that mostly opened directly into air capillaries rather than infundibulae. The exception to this rule was where the parabronchi were adjacent to large blood vessels, in which case, they were the ultimate gas exchange units, well endowed with exchange capillaries (Figure 6). Type II pneumocytes were numerous at the tips of the interatrial septa but were also present at the level of the air capillaries adjacent to the blood capillaries. Cells with mitotic figures were present at the level of the air capillaries, and these were thought to be dividing type II cells. The vast exchange surface of the air capillaries was made by squamous pneumocytes that also participated in the formation of a thin tripartite blood–gas barrier estimated to be about 2 µm (Figure 6).

**Figure 7:**
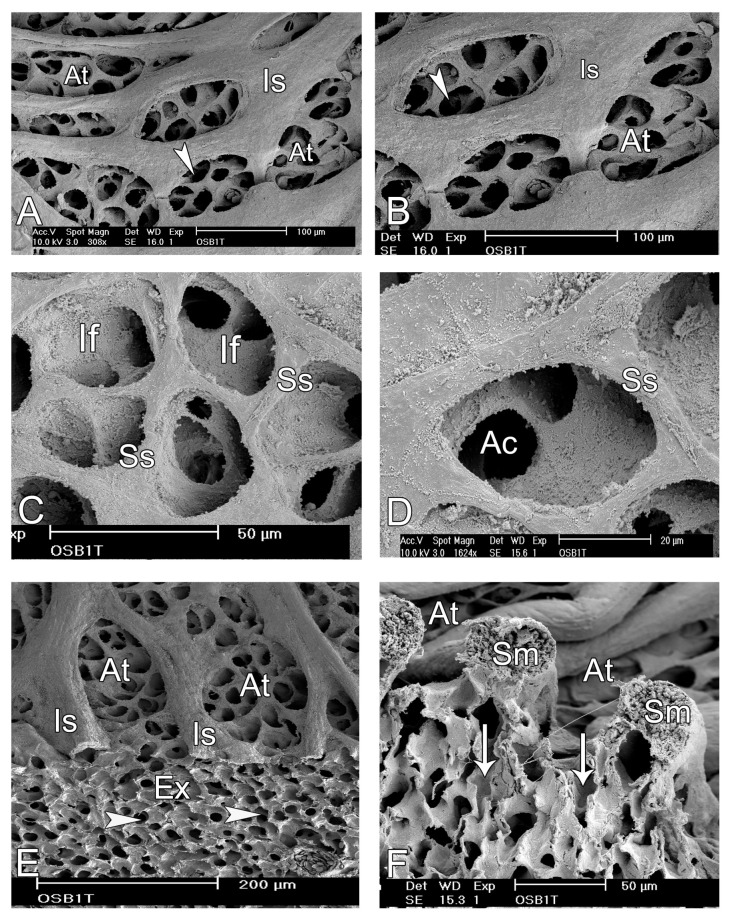
Scanning electron microscope images of the internal aspects of the parabronchi showing the atria, infundibulae, and air capillaries. (**A**,**B**) The atria (At) are shallow and delineation of the infundibulae and air capillaries (white arrowheads) at this magnification is not possible. The prominent interatrial septa (Is) are shown. (**C**,**D**) At higher magnification, the infundibulae (If) appear to emerge from the same level and are separated by secondary septae (Ss). The air capillaries (Ac) emanating from the infundilum, are clearly discernible. (**E**,**F**) Transected portion of a parabronchus (**E**) shows the exchange tissue (Ex), the prominent interatrial septa (Is) separating the atria (At). The white arrowheads indicate transected air capillaries. The prominent septa separating atria (At in (**F**)) are supported by smooth muscle fibres (Sm) below which the air capillaries (white arrows) spread.

The internal aspects of the parabronchi were lined by shallow ovoid atria separated by prominent smooth muscle septae (Figure 7). At low magnification, delineation of the infundibulae and air capillaries was not possible, but at higher magnification the air capillaries were seen to continue from infundibulae, while a few emanated directly from the atria. The air capillaries radiated into the exchange tissue where they branched and anastomosed with their cognates and interacted with the blood capillaries.

**Figure 8:**
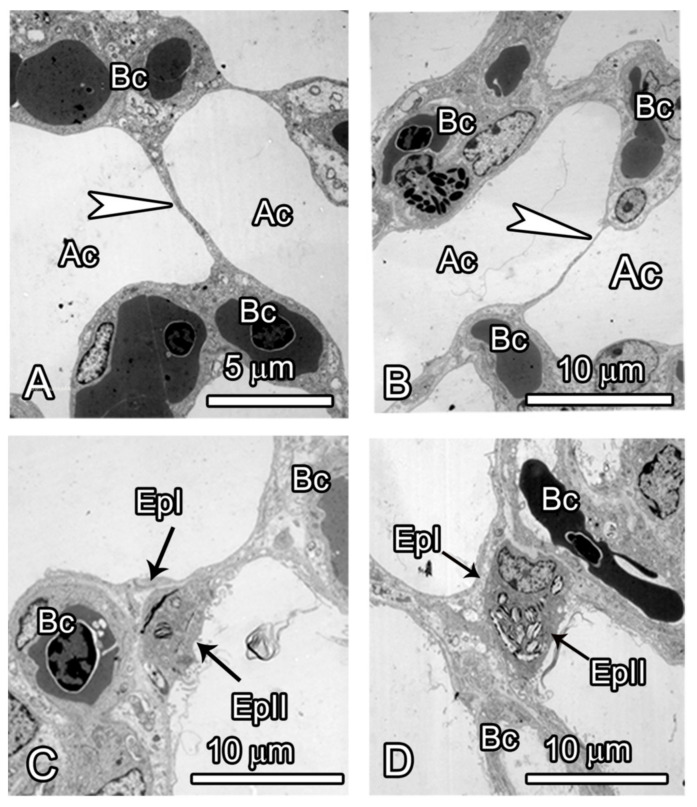
TEM micrographs of the gas exchange tissue of the ostrich lung showing the various categories of epithelial bridges (epithelial plates) that support the blood capillaries. (**A**,**B**) The homogeneous type of plates comprises two leaflets of type I cells (arrowhead) that separate two air capillaries (Ac) but support contiguous capillaries (Bc). Note that these plates can be extremely thin (arrowhead in (**B**)). (**C**,**D**) The heterogeneous epithelial plates comprise a type I cell (EPI) and a type II cell (EPII), These separate neighbouring air capillaries (Ac) but also support blood capillaries. The presence of lamellar bodies means that these plates also participate in the secretion of surfactant.

Further examination of the parenchyma (Figure 8) at the ultrastructural level revealed that blood capillaries were supported by prominent epithelial plates that were of two types: the homogeneous squamous type comprising two leaflets of type I cells and the heterogeneous type consisting of a type I pneumocyte and a type II cell. 

**Figure 9:**
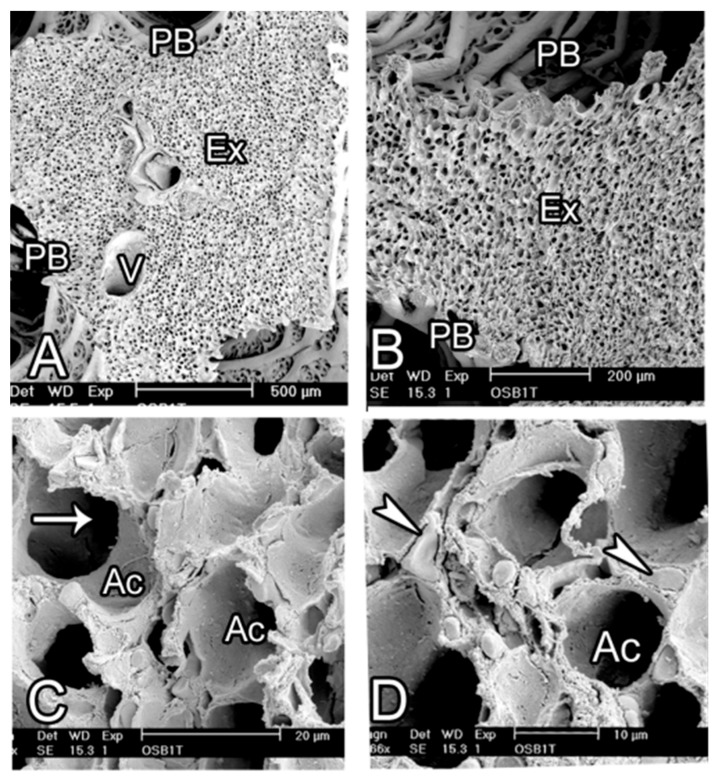
Scanning electron micrographs showing the exchange tissue of the ostrich lung. (**A**,**B**) The parabronchial lumen (PB) is surrounded by the exchange tissue (Ex) that looks homogeneous except where the conducting blood vessels (V) are present. Note that interparabronchial septa are absent. (**C**,**D**) At higher magnification, the septa separating adjacent air capillaries have central blood capillaries packed with erythrocytes (white arrowheads). The air capillaries (Ac) give rise branches (white arrow) that anastomose with their neighbouring cognates.

A bird’s eye view of the parabronchial internal surface revealed the structure of atria, which were separated by very thick interatrial septa. The atria were shallow, ovoid in shape with a longer diameter of about 50–100 µm and numerous openings to the air capillaries and occasionally to infundibulae (Figure 9).

Contiguous infundibulae were separated by secondary septa, and the interatrial septa were supported by thick bundles of smooth muscle (Figure 9).

## 4. Discussion

Despite many contemporary investigations, the structure of the avian lung continues to draw controversies, with conflicting reports appearing in the literature from time to time [13,15]. In the current study, it was observed that the ostrich lung conforms to the general rhomboid shape described for the Chicken [10] although it was much larger. The topographical positioning of the laterodorsal secondary bronchi was similar to that of the Domestic Duck (*Cairinia mochata*) with thin collapsible membranes [11]. Using ultrasound scanning, such secondary bronchi were shown to participate in the propulsion of gases toward the lung parenchyma [19]. The categories, numbers, and spatial disposition of the secondary bronchi are important because they may have significant implications in the implementation of unidirectional airflow [10]. A single morphometric study performed by Maina and Nathaniel [6] noted that the atria are shallow and the lung lacks interparabronchial septa. The significance of such modifications was unclear, but the pulmonary morphometric refinements closely resembled those of highly energetic volant birds [6]. It was previously reported that the neopulmo is very poorly developed, consisting of a few laterodorsal secondary bronchi that arise from the most caudal part of the primary bronchus [6], and their parabronchi are lateral. One new category of secondary bronchi, the posterior secondary bronchi (POSB), which has been missed out previously, was described in chicken [10] and duck [11] lungs. As elucidated elsewhere, the latter category had often been confused with either the laterodorsals or the lateroventrals, but their structure and disposition have now been well explicated [9].

In the ostrich, the categories and the disposition were found to be similar to those of the Chicken and duck, although the numbers were slightly different, with the ostrich having fewer posterior secondary bronchi. These latter categories of secondary bronchi (POSB) have previously been missed by contemporary investigators firstly because they resemble parabronchi (so-called tertiary bronchi) both in structure and size, with the exception that they emerge directly from the mesobronchus and their initial parts have no openings to the atria [10]

The arrangement of the LDSB in the ostrich in close adherence to the intercostal muscles and their collapsible nature are strong pointers to their possible participation in gas propulsion, a phenomenon demonstrated previously in the Domestic Duck [19]. The other categories of the secondary bronchi do not differ much in numbers but are more in the current study than those documented earlier where MVSB were said to be 3, mediodorsal (currently named laterodorsal) were 5, one LVSB but no POSB were recognized [3]. The techniques used to elucidate the types, topography, and categories in the latter study were not elucidated, and the entire study was based on only one juvenile animal [6]. These findings authenticate earlier observations that the structure of the avian lung remains a subject of debate and a lot is yet to be clarified [20,21].

Over a century ago, it was noted that the morphology of the avian lung could only be elucidated by observations of its development [22]. Previously, we reported details on the prehatch development of the ostrich lung, where the preponderance of type II cells at the level of the air capillaries was noted [23]. Indeed, the processes encountered in the development of the ostrich lung closely resembled those of the Chicken lung [24,25,26,27], although in some areas, there were notable processes resembling those of the mammalian lung [25,27]. Earlier, it was demonstrated that the complex structure of the avian lung is best explicated by using several study techniques and also following the structure during development [10]. Indeed, it was in this latter study that a fifth category of the secondary bronchi, the POSB, was discovered. However, despite these new clarifications, many contemporary researchers still use the old nomenclature that generalizes bronchi into ventral, lateral and dorsal [17,28]. Recently, the many discrepant names of the various avian bronchi have been highlighted [17]. 

The structure of the ostrich lung exchange tissue differs fundamentally from that of the ‘archetypical avian lung’ in that the rigid interparabronchial connective tissue septa are lacking, and the type II cells are present down to the level of the air capillaries [6] (this study), a phenomenon found in other ratites for which data exist [29].

Detailed studies on the lung of ostrich are lacking, and although morphometric analyses have been carried out, they were inconclusive since they were based on one individual [6]. Three-dimensional reconstruction of the gas exchange tissue in the avian lung has been attempted, but conclusive inferences have been elusive due to limitations associated with resolution at the light microscopic level [12,14,16].

Earlier studies described the parabronchi to be numerous anastomosing small tubes that leave the secondary bronchi and were, as such, referred to as tertiary bronchi. However, some researchers reported that some parabronchi emanated directly from the main bronchus [30]. The latter were hitherto unrecognized posterior secondary bronchi, described later [10]. The final category of secondary bronchi is known as saccobronchi or bronchi recurrentes [30] and are important in the conveyance of gases between the lung parenchyma and the air sacs, facilitating unidirectional airflow. 

The use of different casting materials in the study of the avian lung is advantageous because it allows both flexibility and improved resolution of the structures under investigation [31]. Silicon rubber is pliable and allows manipulation of the casts of the tubules so that fine details can be observed [32]. Resin casting, on the other hand, results in rigid structures that depict the in situ situation, and combined with sputter-coating and scanning electron microscopy, can resolve very fine details [31]. Observation of the mesobronchial lumen on longitudinally bisected intrapulmonary primary bronchus reveals virtually all the openings to the secondary bronchi apart from those that occasionally emerge from other secondary bronchi [10]. Studies on the parabronchial unit in the ostrich lung using 3D reconstruction have shown that the air capillaries (ACs) and the blood capillaries (BCs) are not straight blind-ending tubes but are anastomosing structures that interact both in cross-current and countercurrent orientations, what increases gas exchange efficiency [12].

The limitations of a single investigative technique, however, cannot be overemphasized. In contemporary studies on the avian lung, the actual relationship between the ACs and BCs has been elucidated to appreciable details. At the AC-BC interface, a cross-current arrangement is predominant [33,34]. In the current study, some atria have been shown to be the terminal gas exchange units; most air capillaries emanate directly from the atria, and infundibulae are few, corroborating previous studies on the ostrich lung [6].

The avian lung is generally noted to be non-compliant with a strong albeit very thin blood–gas barrier (BGB). These advantages for gas exchange have been attributed to the support of avian pulmonary capillaries by the surrounding air capillaries. This arrangement was made possible by the adoption of the flow-through system of ventilation in birds as opposed to the reciprocating pattern in mammals [35]. In the ostrich lung, the epithelial plates were of two types due to the presence of two cell types at the air capillary level, namely, the homogeneous and the heterogeneous type. In the former case, the leaflets were made of two type I cells, while the heterogeneous type comprised a type I cell and a type II cell. The junctions of the bridges with the capillary walls show thickening of the epithelial cells and an accumulation of extracellular matrix [36,37,38]. The support of the pulmonary capillaries may also be explained by an interdependence mechanism whereby the blood capillaries are linked to a rigid assemblage of air capillaries [37]. Besides the support rendered to the blood capillaries in the ostrich lung, the heterogeneous type of plates participates in surfactant production and may act as reservoirs of stem cells for the gas exchange epithelium.

The reason why birds can be so highly energetic yet also have such apparently fragile capillaries is the mechanical support provided by the dense packing of rigid air capillaries around the blood capillaries in the gas-exchanging region of the lung [38]. Generally, the strength of the avian lung can be explained by the tension integrity arrangement of the various components that form this gas exchanger [39], in which the hexagonal arrangement of the parabronchial unit and the presence of tough connective tissue-reinforced interparabronchial septa play a crucial role [38]. The ostrich lung studied here lacks the interparabronchial connective tissue septa and, as such, the hexagonal arrangement is not apparent.

## 5. Conclusions

In conclusion, we observe that although the general design of the ostrich lung resembles that of other avians, the presence of numerous type II cells down to the air capillaries and the absence of tough interparabronchial tissue septa may be a pointer to some degree of compliance, but currently, no investigations have tested this possibility.

## Figures and Tables

**Table 1:** Techniques applied in the study and respective numbers of animals used.

Technique	Animal No.	Age	Sex	Results Accomplished
Macroscopic and Microscopic examination	1.	Adult	Male	Enumeration of the secondary bronchi; study of the relationship between the secondary bronchi, the intercostal muscles and the ribcage; semithin sections, SEM and TEM micrographs.
	2.	Adult	Female	
	3	Adult	Male	
Macroscopic examination	4	Adult	Male	Enumeration of the secondary bronchi.
	5 *	Adult	Female	Study of the rib cage skeleton and its relationship to the topography of the lung
Macroscopic and Microscopic examination	6	Chick	ND	Enumeration of the secondary bronchi; semithin sections, SEM and TEM micrographs.
	7	Chick	ND	
Silicon rubber casting	8	Chick	ND	Study of the 3D arrangement of secondary bronchi
	9	Chick	ND	
	10	Chick	ND	

NB: Enumeration of secondary bronchi was carried out on opened up mesobronchi and entailed counting of their respective openings. This was carried out both for the chicks and adults. ND = sex not determined; * Specimen number 5 was a skeleton of a female ostrich mounted in the Department of Veterinary Anatomy and Physiology.

**Table 2:** Categories and numbers of the secondary bronchi in the ostrich lung. The abbreviations for the secondary bronchi are as follows: MVSB—medioventral; LDSB—laterodorsal; LVSB—lateroventral, POSB—posterior. L and R denote the left and right lungs, respectively.

Animal. No.	MVSB		LDSB		LVSB		POSB	
	L	R	L	R	L	R	L	R
1	4	4	8	10	5	5	24	22
2	4	4	8	10	4	6	18	20
3	4	4	8	8	4	6	20	22
4	4	4	8	8	5	5	18	20
5	4	4	8	10	5	5	20	18
6	5	5	8	9	5	5	20	6
Mean	4.2	4.2	8	9.2	4.6	5.3	20	20.6
SD	0.37	0.37	8	0.9	0.47	0.47	2	1.5
Range	4–5	4–5	8–8	8–10	4–6	5–6	20–24	18–22

**Table 3:** Comparison of the numbers of secondary bronchi in the ostrich lung with those of the Chicken and duck lungs. For the ostrich lung, averages for both the left and right lungs have been calculated from Table 2 above.

	Chicken		Duck		Ostrich	
CATEGORY	Range	Mean	Range	Mean	Range	Mean
**MVSB**	4–4	4	4–4	4.4	4–5	4.2
**LDSB**	6–10	9.5	6–10	8.2	8–10	8.1
**LVSB**	1–3	1.4	2–4	3	4–6	4.7
**POSB**	20–60	37	38–44	40	18–24	20.3

Sources of data: chicken—Makanya and Djonov [10]; Duck—Makanya et al. [11]; Ostrich: this study. The abbreviations are as for Table 2 above.

## Data Availability

The data presented in this study are available on request from the corresponding author. The data are not publicly available due to the fact that some are part of ongoing reseearch.
